# An Overview of Organs-on-Chips Based on Deep Learning

**DOI:** 10.34133/2022/9869518

**Published:** 2022-01-19

**Authors:** Jintao Li, Jie Chen, Hua Bai, Haiwei Wang, Shiping Hao, Yang Ding, Bo Peng, Jing Zhang, Lin Li, Wei Huang

**Affiliations:** ^1^Frontiers Science Center for Flexible Electronics, Xi'an Institute of Flexible Electronics (IFE) and Xi'an Institute of Biomedical Materials & Engineering, Northwestern Polytechnical University, Xi'an 710072, China; ^2^Key Laboratory of Intelligent Computing and Signal Processing of Ministry of Education, School of Electronics and Information Engineering, Anhui University, Hefei 230601, China; ^3^38th Research Institute of China Electronics Technology Group Corporation, Hefei 230088, China; ^4^College of Biomedical Engineering, Sichuan University, Chengdu 610065, China; ^5^Key Laboratory of Flexible Electronics (KLOFE) and Institute of Advanced Materials (IAM) Nanjing Tech University (NanjingTech), Nanjing 211800, China

## Abstract

Microfluidic-based organs-on-chips (OoCs) are a rapidly developing technology in biomedical and chemical research and have emerged as one of the most advanced and promising *in vitro* models. The miniaturization, stimulated tissue mechanical forces, and microenvironment of OoCs offer unique properties for biomedical applications. However, the large amount of data generated by the high parallelization of OoC systems has grown far beyond the scope of manual analysis by researchers with biomedical backgrounds. Deep learning, an emerging area of research in the field of machine learning, can automatically mine the inherent characteristics and laws of “big data” and has achieved remarkable applications in computer vision, speech recognition, and natural language processing. The integration of deep learning in OoCs is an emerging field that holds enormous potential for drug development, disease modeling, and personalized medicine. This review briefly describes the basic concepts and mechanisms of microfluidics and deep learning and summarizes their successful integration. We then analyze the combination of OoCs and deep learning for image digitization, data analysis, and automation. Finally, the problems faced in current applications are discussed, and future perspectives and suggestions are provided to further strengthen this integration.

## 1. Introduction

The most widely used experimental models in biological research are cell-based and animal models; however, both these models have many limitations. Traditional cell-based models lack essential features, such as complex multiple cultures, physiological microenvironments, and tissue mechanical forces [1]. Animal models, although regarded as the current gold standard in many biological studies, have problems such as high cost, ethical issues, low throughput, and interspecific differences, which significantly limit the progress of drug development and other biological research [2, 3].

Microfluidic-based OoC technology is proposed to fill the gap between traditional two-dimensional (2D) cell culture and animal models and to gradually replace animal studies [4]. As a product of the progressive development of microfluidic technology, OoCs combine microfluidic technology with cell biology; they faithfully mimic the physiological microenvironment of the *in vivo* target organs. These novel *in vitro* biological models can replicate the local characteristics of a disease and control the environmental parameters of cell survival, making them a cost-efficient and high-throughput platform for biological research. Polydimethylsiloxane (PDMS) and poly(methyl methacrylate) (PMMA) are the most commonly used materials for the fabrication of OoC devices. Owing to the transparent nature of these materials and its high compatibility with fluorescence microscopy, OoC applications usually generate a large number of images, leading to a large amount of image-based data. This has traditionally been accumulated and processed by manual methods, which are typically inefficient [5].

The utilization of automatic and intelligent data analysis systems will further enhance the development of OoCs in various biomedical applications. Deep learning [6] is the most representative research field in artificial intelligence (AI) [7]. The application of deep learning to OoCs offers a powerful tool for the exploration and analysis of the massive image-based data generated by OoC approaches, which consequently enhances the automated level of OoCs. Riordon et al. reviewed the integration of deep learning with microfluidics [8]. However, in the field of OoCs, no review focused on this novel concept to date. Therefore, a timely and comprehensive summary of the applications of deep learning in OoC studies will promote the development of this technology and facilitate research in both fields.

**Figure 1:**
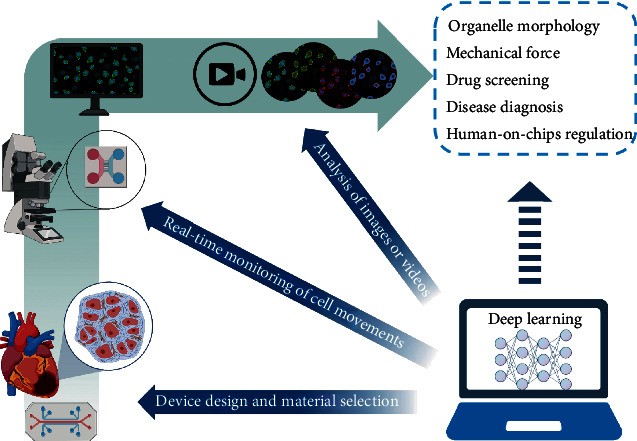
Integration of deep learning with organs-on-chips (OoCs). Deep learning has been applied to device design, real-time monitoring, and image processing in OoCs. In the future, it may be further applied to organelle tracking, mechanical force mimicking, drug screening, rare disease diagnosis, and human-on-chip regulation (created with http://BioRender.com).

This review provides an in-depth discussion of the integration of deep learning and OoCs (Figure 1). Following the introduction of the basic concepts of OoCs and deep learning, we review deep learning as a multifunctional data analysis tool for biomedical applications, including cell identification, localization, tracking, and image segmentation. Finally, we discuss future directions for the application and integration of deep learning in the field of OoCs.

## 2. Emergence of OoC Technology

Replicating the human physiological system is extremely important for the pharmaceutical industry to predict drug efficiency, pharmacokinetics, and toxicity [9]. Animal models are currently the gold standard for many biological studies and can provide the most accurate predictions. However, the associated high costs, low throughput, and ethical issues limit the application of animal models to the early stages of drug discovery [10]. In addition, interspecies differences are an insurmountable gap between animal models and humans; thus, the experimental results for some disease models [11] and drug efficacy studies [12] have deviated from those of humans. For *in vitro* models, most biological studies have relied on two-dimensional (2D) cell cultures [13]. Despite the value of this model, it does not adequately reconstruct the *in vivo* cell microenvironment or simulate the complex physiological functions of human organs. To solve this problem, three-dimensional (3D) cell culture models have emerged and provide several enhancements compared to traditional 2D cell culture, such as improvement of the expression of differentiation functions, tissue structure, signal capture, and drug response sensitivities [4]. However, even the most effective 3D models are still unable to perfectly reproduce the complex cell–cell interaction, spatial configuration of different types of cells, and tissue mechanical forces of human organs.

**Figure 2:**
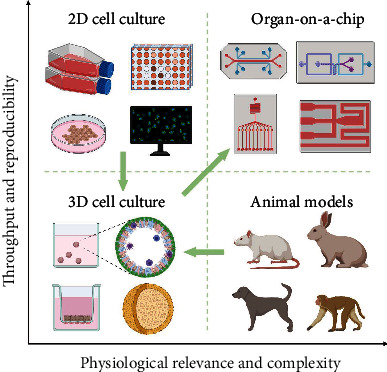
Emergence of OoC technology provides a strong connection between animal models and traditional *in vitro* models. It considers the physiological relevance and complexity as well as throughput and reproducibility (created with http://BioRender.com).

Recent research in microfluidic systems and cell biology has created novel engineered microphysiological systems, OoCs [1]. These *in vitro* models provide tissue mechanical force and a controllable microenvironment, allowing the reconstruction of fundamental features of the target organ/tissue. Therefore, the introduction of OoCs has bridged the gap between oversimplified 2D cell culture and expensive animal models, providing an efficient and energy-saving biological research platform (Figure 2).

### 2.1. Microfluidic Technology

Microfluidics are miniaturized systems with specific morphological and positional structures. Their width and height scales are both between 100 nm and 100 *μ*m [14]. The reaction time in microfluidic systems is much shorter than that in conventional instruments because the small system rapidly diffuses molecules [15]. The implementation of microfluidics is inextricably linked to the rapid development of photolithography and inkjet printing technologies. At the same time, researchers have designed pumps and valves capable of controlling and manipulating fluid flow [14]. Thus, microfluidic systems have the advantages of fast operation and small size and can manipulate fluids on the microscopic scale, enabling physiological fluid shear and pulsating flow patterns. Small-size microfluidic systems use less reagent than traditional flow control platforms and are thus perfect tools for high-throughput screening [15, 16]. In the past two decades, microfluidics have been successfully used in various biological applications such as fast cell sorting [17], cell biochemistry analysis [18], biomaterial screening [16], and OoCs [1, 19].

### 2.2. OoC Technology

**Figure 3:**
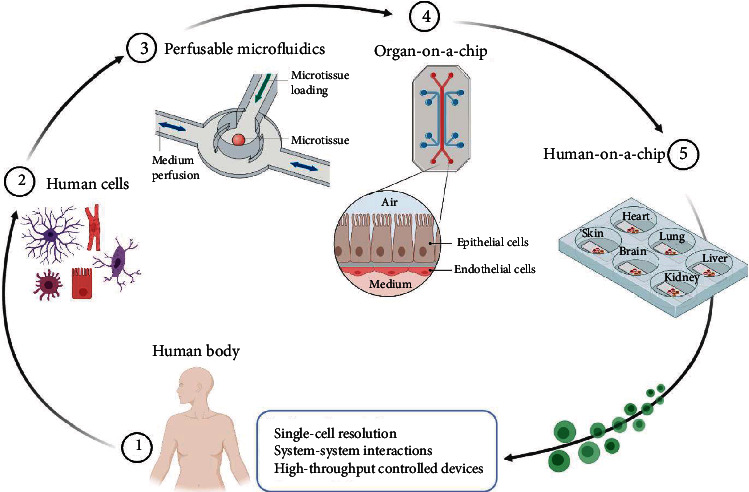
Integration of microfluidic technology, biomaterials, and cell biology results in an advanced *in vitro* OoC system. Cells from a human body are extracted (2) and placed in perfusable microfluidic devices (3) to make OoCs (4). Multiple OoCs connected together results in a human-on-a-chip system, (5) which ultimately will faithfully replicate the key functions of the human body and therefore holds great potential for use in drug discovery and pathological research (created with http://BioRender.com).

Owing to the intrinsic characteristics of microfluidic technology, such as miniaturization, highly controlled flow systems, and flexible device designs, the integration of microfluidic technology, biomaterials, and cell biology has resulted in an advanced *in vitro* OoC system (Figure 3). Compared to conventional *in vitro* cell models, OoCs can accurately control parameters such as the chemical concentration gradient [20], tissue mechanical force [21], cell spatial configurational culture [22], multiple-cell coculture [23], and organ–organ interaction [24], in order to replicate the complex structures, microenvironments, and physiological functions of human organs. In addition, physiological barrier models based on OoCs accurately simulate the delivery and penetration of compounds *in vivo* [25]. In recent years, the precision of OoCs has increased dramatically, allowing assays to be performed on single cells and enabling high throughput, with thousands of simultaneous quantitative analyses at single-cell resolution [26].

**Figure 4:**
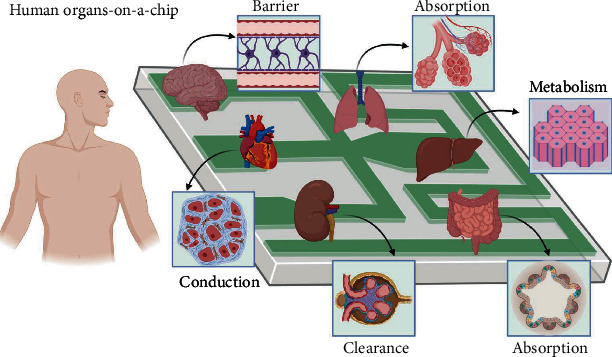
Some successful applications of OoCs and their corresponding functions (created with http://BioRender.com).

After rapid developments in recent years, researchers have replicated several human organs in OoCs (Figure 4). Ho et al. successfully mimicked the structure of liver lobules by patterning liver cells and epithelial cells on a circular PDMS-based microfluidic chip to simulate the lobular structure of the liver [27]. Huh et al. designed a double-layer lung-on-a-chip, which deformed the PDMS membrane using a vacuum pump to simulate the expansion and contraction of the alveolar wall during respiration [28]. This work was regarded as a landmark study concerning OoC technology. Kim et al. utilized a similar design to simulate the expansion of human intestinal peristalsis [29]. Jang et al. replicated the proximal tubular structure of the kidney by introducing fluid shear in a bilayer OoC [30]. Ren et al. also constructed a capillary endothelial barrier using two parallel microcolumn arrays to accurately mimic the structure and function of myocardial tissue [31]. By combining polymer chemistry and OoC technology, we recently reported a blood–brain barrier (BBB)-on-a-chip with simulated BBB function. We successfully evaluated the permeability of small-molecule drugs and monitored the endocytosis and transcytosis of nanomaterials in the endothelium [32, 33]. Furthermore, collaboration between research institutions and pharmaceutical companies has brought OoCs to a practical stage. A kidney-on-a-chip has been successfully applied for drug screening [34]. In addition, Johnson & Johnson plans to conduct drug trials using the human thrombus simulation OoCs developed by Emulate and use the liver-on-a-chip to test the hepatotoxicity of drugs [35].

As can be seen from this discussion, OoCs have been developed for the major organs of the human body; an increasing number of organ models have also been developed for other less studied organs and tissues, such as muscle models [36], bone models [37], tissue models [38], mammary gland models [39], skin models, and others [40, 41].

## 3. Deep Learning

**Figure 5:**
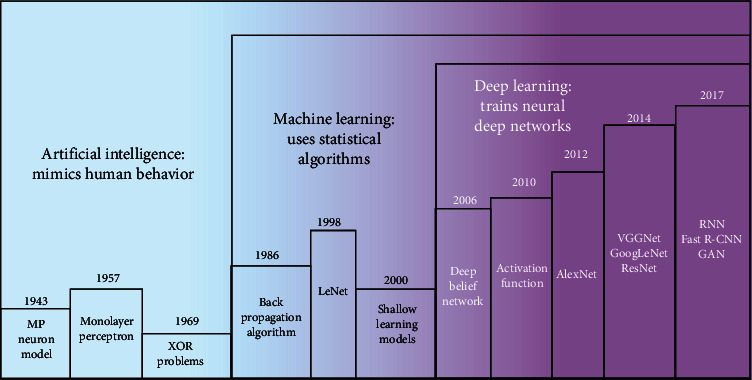
Visual relationship among AI, machine learning, and deep learning.

In recent years, with more powerful computing performance of graphics processing units (GPUs) [42] and the improvement of big data acquisition capabilities, deep learning has led to the creation of state-of-the-art benchmarks in many industries and has become the preferred intelligent technology for engineering applications. It has been widely used in many fields, such as natural language processing [43], speech recognition [44], and computer vision [45]. Based on the relation between three current topics of interest in computer science, AI [7], machine learning [46], and deep learning [6], we provide an in-depth analysis of the development context of deep learning (Figure 5). Machine learning is a common technical means to realize AI, and deep learning is a type of machine learning algorithm. AI is applied to mimic human thinking, perceive the environment, and take action to achieve goals. Machine learning refers to choosing the most appropriate algorithm based on a large amount of historical data so that machines can learn inherent regular information to effectively solve practical problems. There are a vast array of algorithms in machine learning, the most widely used of which is neural network-based deep learning. The behavior of neural network-based deep learning mimics many characteristics of the human brain; it also includes some of the basic functions of the brain by simulating the structure and character of the cerebrum.

**Figure 6:**
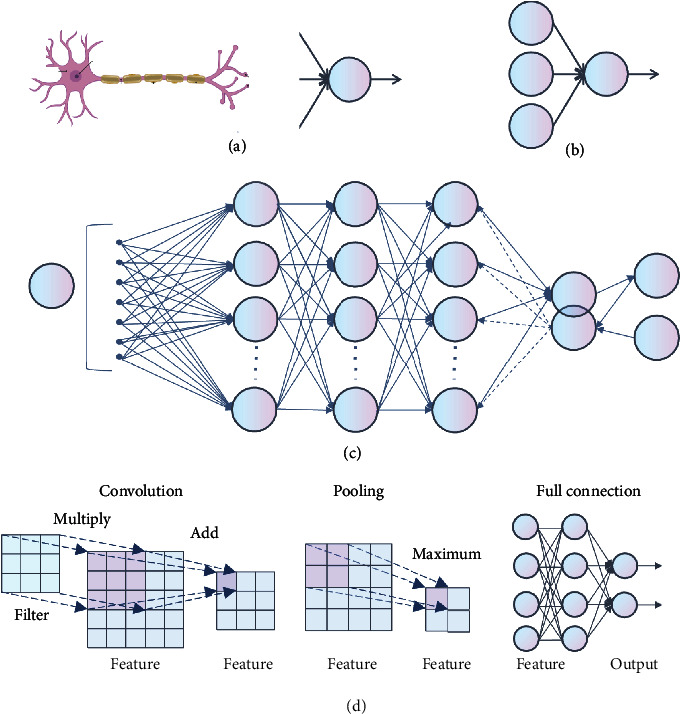
In-depth analysis of the development context of deep learning. (a) Biological neuron and M-P model. To simplify the model and facilitate expression, the model ignores complex factors in biology. (b) Monolayer perceptrons. (c) Back propagation algorithm. (d) Convolution neural networks.

In 1943, McCulloch and Pitts jointly proposed the McCulloch–Pitts (M-P) model (Figure 6(a)) and developed the theory of neural networks, which provided a foundation for the growth of machine learning [47]. In 1957, neural networks were first developed, and Rosenblatt et al. established the concept of a monolayer perceptron (Figure 6(b)), which became the first neural network model [48]. It is a simple neural network that linearly divides data into two categories. The input is the eigenvector of the instance, the output is the category of the instance, and the binary values of +1 and -1 are used. However, it was not until 1969 that Minskey and Papert demonstrated that the perceptron was incapable of facing linear inseparable problems similar to XOR problems; this led to the development of machine learning over a ten-year period of research [49].

In 1986, as the originator of deep learning, Rumelhart et al. proposed the famous back propagation (BP) algorithm (Figure 6(c)), which can solve linear inseparable problems such as XOR and thus promoted the wave of research into the second generation of neural networks [50]. An error between the actual and reference values became apparent when training the network. They then used the gradient descent method to reduce this error as much as possible. After forward propagation, the gradient of the error relative to the model parameters was calculated. This gradient was propagated back through the method of gradient descent to modify the weight of synaptic connections among different neurons, gradually finding the best combination of weights and deviations to reduce the error to a minimum and improve the performance of the model.

**Figure 7:**
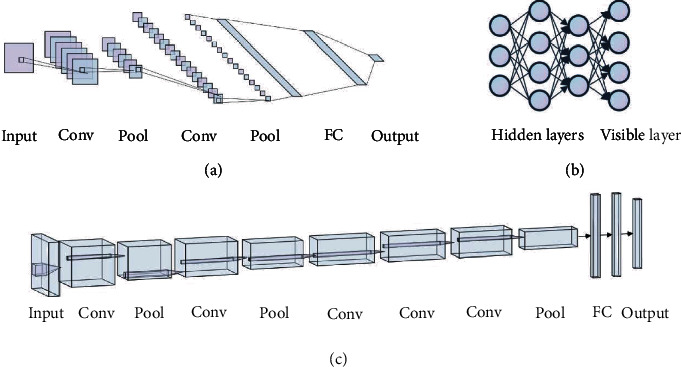
Some typical deep learning networks. (a) Architecture of LeNet. Each square is a feature map, and the weights of each set of squares are constrained to be identical. (b) Architecture of DBN. The DBN consists of several hidden layers and a visible layer, with connections between the layers, but not between the units of each layer. The hidden layers are trained to capture the correlations of data displayed in the visual layer. (c) Architecture of AlexNet. It is similar to LeNet but replaces large convolution with some convolution (Conv) layers, which means it is deeper than LeNet. In addition to this, it uses ReLU as the activation function and considerably more data than LeNet.

BP has become the most commonly used optimization algorithm for multilayer perceptron training. In addition, another deep learning pioneer, Lecun et al., proposed convolutional neural networks (CNNs) to successfully realize handwritten digit recognition [51]. This was the world's first CNN architecture, the famous LeNet network (Figure 7(a)). A CNN architecture typically consists of several convolution layers closely followed by pooling layers and a fully connected (FC) layer (Figure 6(d)). In the convolution layer, the feature map of the previous layer and a filter from the upper left corner create a convolution that multiplies the corresponding numbers and then adds them. The filter slides smoothly until all the features are calculated; thus, the output forms a feature map of this layer. The pooling layer, which contributes to aggregating features and reducing dimensions, is positioned between two convolution layers. It divides the input data into different regions, and the image resolution in each region is reduced through pooling operations. The main purpose of the FC layer is to connect the features one by one to the marker space. All its connections are tightly linked to those of the previous layer, thus transforming the multidimensional output into a one-dimensional vector and achieving classification. However, owing to the disappearance of the gradient, the limitation of the number of training samples, the lack of computing power, and the introduction of shallow learning models such as support vector machines (SVM) [52], logistic regression (LR) [53], decision tree [54], and the naive Bayesian model (NBM) [55], the neural network has not been widely applied and promoted, and research in this area has reduced.

In 2006, Hinton et al. proposed deep belief networks (DBNs) (Figure 7(b)), which effectively shortened the training time of deep neural networks and alleviated the problem of gradient disappearance caused by the BP algorithm [56]. In addition, a new activation function, the rectified linear unit (ReLU), was constructed. Experiments showed that using ReLU could suppress the vanishing gradient problem [57].

In 2012, deep learning became a popular research topic. In the ImageNet Large Scale Visual Recognition Challenge (ILSVRC), Krizhevsky et al. built a multilayer convolutional neural network, AlexNet (Figure 7(c)), to achieve an image classification error rate that was significantly reduced from the previous lowest of 26% to 15% [58]. This record-setting performance surprised the entire industry and stimulated research interest in neural networks again. Numerous models based on the deep CNN architecture are emerging, and many impressive results have been achieved [59]. Representative CNN architectures include VGGNet [60], GoogLeNet [61], and ResNet [62].

In addition to CNNs, many other branches of research in the field of deep learning have been developed recently, including sequence prediction represented by recurrent neural networks (RNNs) [63] and transformers [64], image generation represented by generative adversarial networks (GANs) [65], object detection represented by Faster R-CNN [66], YOLO [67], semantic segmentation represented by U-Net [68], and DeepLab [69].

In recent years, deep learning has been successfully applied to commercial applications by various manufacturers; the applications include Google Translate, Apple's voice tool Siri, Microsoft's Cortana personal voice assistant, and Ant Financial Smile to Pay [70]. Most importantly, deep learning can potentially help in the mitigation of diseases such as the coronavirus disease (COVID-19), which has resulted in a global pandemic over the past two years. Deep learning technology is likely to play a large role in the identification of epidemiological characteristics across many countries and to enable the exploration of the development trend of pandemics, thus providing a basis for creating control measures. In addition, research teams in an increasing number of industries are incorporating deep learning in the exploration of research and commercial applications, such as medical diagnosis, pandemic tracking and prediction, industrial intelligent manufacturing, autonomous driving, and virtual reality.

## 4. OoCs and Deep Learning Integration

OoCs and deep learning are frontier disciplines in biomedical engineering and AI, respectively. In this section, we first introduce the application of deep learning to microfluidics and then extend it to OoCs (Table 1). Because the combination of these two disciplines is not widely explored at present, we also provide some perspective on the application of deep learning to the following aspects of OoCs: prediction, target recognition, image segmentation, and tracking.

### 4.1. Deep Learning in Microfluidics

The development of deep learning has resulted in great progress in microfluidics research and has led to a new generation of microfluidic platforms with extensive functions. Furthermore, the applications of deep learning in microfluidics have allowed researchers to observe phenomena that were difficult to capture in the past. We have divided these applications into two categories: device parameters and images.

#### 4.1.1. Deep Learning in Device Parameters

**Figure 8:**
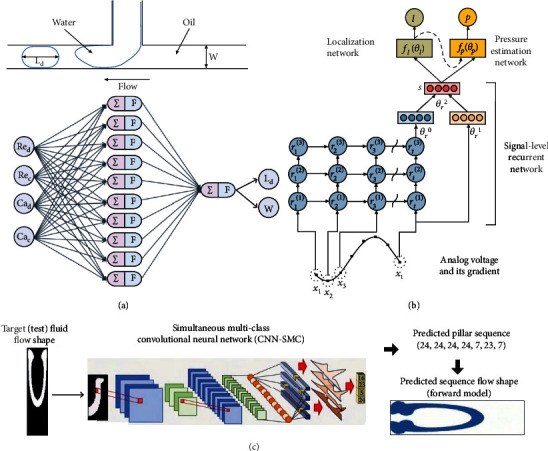
Deep learning in device parameters. (a) Formation mechanism of microdroplets in a microfluidic T-junction. The dispersed phase is perpendicular to the lateral channel. Two syringe pumps supply and control two fluids. The neural network below consists of 10 neurons in the hidden layer (reproduced with permission from Ref. [71]). (b) Hierarchical signal-level recurrent network, which could concurrently learn to forecast pressure and location. Reproduced with permission from Ref. [72]. (c) Workflow for the trained deep learning network (reproduced with permission from Ref. [73]).

The application of microfluidic devices in emulsion production has resulted in advantages such as reduction of reagents, product emulsions with a narrow molecular weight distribution [74, 75], and high-value-added products [76]. Changing and grouping all the parameters (i.e., flow rate, viscosity, two-phase surface tension, and microchannel diameter) can affect the T-junction microdroplets. Mahdi et al. used these dimensionless parameters as inputs to a neural network [71]. After cyclic training in hidden layers, the number and interconnectivity of the neurons were determined. Finally, the network output the predictions of the dimensionless length *L*_*d*_ and junction width *w* of the microdroplet (Figure 8(a)).

Soft sensors [77] used in microfluidics are composed of highly deformable polymers. They are used in elastomeric actuators [78], soft wearable robotic devices [79], and soft robotic grippers [80]. However, compared with traditional sensors, the main disadvantages of microfluidic soft sensors are the nonlinearity and hysteresis of the response. Han et al. used a hierarchical signal-level recurrent network to characterize a microfluidic soft sensor that could identify the pressure and location of the stimulus (Figure 8(b)) [72]. In a microfluidic channel filled with liquid metal, the simulated voltage varied with the pressure and location of the channel. Initially, the network aggregated time-series information with three hidden layers and transformed the sequential input data into a representation. The network then identified the locations at which the sensor was being pressed. Finally, the pressure estimation network predicted the magnitude of the pressure corresponding to the sensor outputs. The obtained time-series-related data were entered into the RNN for model training. The model could identify the pressure and location of the stimulus along the channel.

Microfluidic chip design and fluidic modeling require numerous calculations and a knowledge of hydromechanics, which may be an obstacle for researchers with a biomedical background. The most common approach is to make a large number of interactive and intuitive choices among a wide range of design options, which is a time-consuming and labor-intensive task. Stoecklein et al. answered the question “what kind of geometry produces an ideal microfluidic shape?” using deep learning (Figure 8(c)) [73]. A CNN architecture used target flow shapes from a testing dataset and predicted the flow shape. Compared with the original network, neural networks could independently select the best option. The intelligent sampling of this type of data can greatly improve performance and enable effective prediction outside the training range.

#### 4.1.2. Deep Learning in Images

The algorithmic rules for the recognition of cell images are mostly based on mathematical principles and statistical theorems, which are a part of traditional machine learning. For example, a peripheral blood smear image consists of three types of cells: red blood cells, white blood cells, and platelets. The latter two are morphologically different from red blood cells. Marker-controlled algorithms have been used to separate white blood cell nuclei in microscopic images [81]. In addition, there are morphological and threshold selection techniques [82], cluster segmentation algorithms and rule-based methods [83], mathematical morphological and particle-size measurement methods [84], and grayscale thresholding methods [85]. These can also achieve cell recognition. The combination of traditional machine learning with microfluidics has enabled single-cell lipid screening [86] and cell counting [87].

However, owing to the complexity of the multiple types of data generated by the highly parallel operation of microfluidics, traditional machine learning is no longer sufficient to satisfy the requirements of researchers. The application of deep learning, which is a popular method for processing large amounts of data with high efficiency, is appropriate owing to advances in technology. Compared with traditional machine learning, the advantage of the integration of deep learning in microfluidics is clear: it can be used to train complex neural networks to obtain the internal features of the data and improve the efficiency of experiments using large, high-dimensional datasets.

Deep learning has been used to identify and classify mobile cells in microfluidic channels using an RNN architecture. Cell feature vectors (e.g., roundness, circumference, and major axis length) obtained by various imaging modalities have been fed into the networks, and the class of diagnosed cells (e.g., leukocytes and colon cancer cells) was identified. Label-free cell classification was achieved by Singh et al. using the aforementioned approach [88]. Chen et al. used a quantitative time delay microscope to obtain rich cell characteristic data and used deep learning methods to achieve cell classification [89]. The accuracy of this method exceeded that of traditional machine learning. San-Miguel et al. used microfluidic techniques to capture the localization of *Caenorhabditis elegans* arrays and imaged their synaptic punctum patterns [90]. This work identified subtle differences between mutations by feeding the measured data into an RNN architecture, revealing their hidden genetic differences.

**Figure 9:**
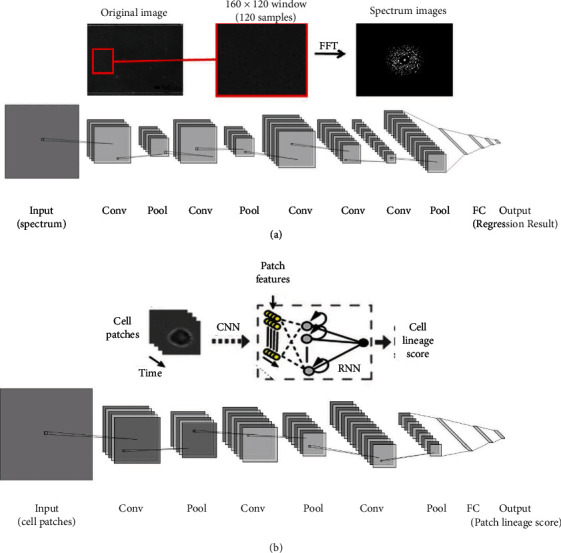
Deep learning in images. (a) CNN structure estimated bacterial growth in microfluidic channels (reproduced with permission from Ref. [91]). (b) CNN architecture combined with an RNN architecture, which could use the temporal information of a single-cell track to choose the lineage of a stem cell's progeny automatically (reproduced with permission from Ref. [92]).

Most existing measurement methods are not suitable for microfluidic equipment with small sample volumes as the level of bacteria in the channel needs to be measured during culturing. Hence, Kim et al. developed an image-based method to assess the growth status of bacteria in microfluidic channels [91]. In this study, bacteria were cultured in a microfluidic device with liquid and agar gel media in two separate channels. Time-lapse images were captured, and a fast Fourier transform (FFT) was used to detect variable frequencies of the images. The experimentally obtained images were used as input to the CNN architecture (Figure 9(a)). Using this model, the level of *Pseudomonas aeruginosa* was successfully obtained and the bacterial growth in the microfluidic channels was quantified.

In addition, a combination of CNN and RNN architectures can be applied when complex image input and temporal information need to be achieved. For example, cell differentiation changes the intracellular molecular properties of original stem cells, resulting in changes in their morphology and motility. Buggenthin et al. combined CNN with RNN architecture to predict single-cell lineages when identifying hematopoietic lineages (Figure 9(b)) [92]. The model first used a CNN architecture to extract local abstract features of stem cells under bright field images, and the vector of outputs indicated whether they were similar to certain cell patches. Then, the vector of outputs was fed into an RNN with a bidirectional long short-term memory network (LSTM) architecture to model cell dynamics. The effect of temporal information of the cells in the video was analyzed, and individual cells were classified as belonging to a certain lineage. This hybrid method improved the prediction ability of the model compared with that of CNN; moreover, it predicted the pedigree selection of primary hematopoietic cells. Using a similar approach, it would also be possible to identify the shape of the cells and analyze the movement morphology.

### 4.2. Deep Learning in OoCs

In this section, we discuss various applications of deep learning in OoCs. By discussing examples of each type of application in detail, the power and versatility of the integration of deep learning with OoCs were illustrated.

**Figure 10:**
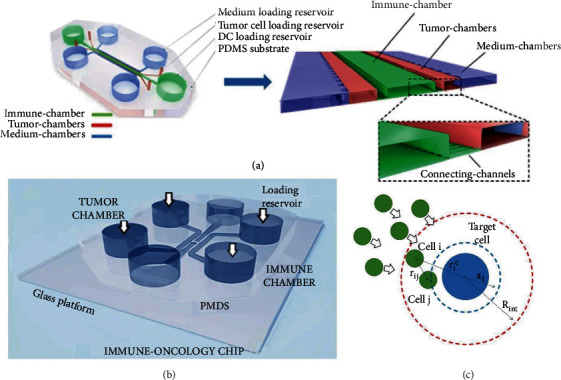
Deep learning in OoCs. (a) 3D schematic device for real-time monitoring of cell interactions (reproduced with permission from Ref. [94]). (b) General scheme of the tumor-on-a-chip consists of six reservoirs for culture medium replacement and four chambers for cell culture (reproduced with permission from Ref. [96]). (c) Definition of the stochastic particle interaction model. The physical interactions among cells were performed through repulsion–attraction exchanges. For the immune cell–cancer interaction, they imposed an attraction in the proximity of the target cell (reproduced with permission from Ref. [97]).

Dendritic cells play a critical role in the recognition of tumor cells by absorbing tumor antigens and presenting them to T cells. The effectiveness of immune therapy therefore relies heavily on the interaction between the tumor and dendritic cells in the tumor microenvironment to induce an effective antitumor response [93]. Parlato et al. reconstructed an interconnected 3D immune cell–tumor ecosystem by combining OoCs with advanced microscopy techniques (Figure 10(a)) [94]. The device was composed of a central immune chamber, which was interconnected with the tumor chamber via an array of microchannels. On this tumor-on-a-chip device, CellHunter [95], an unsupervised cell tracking analysis algorithm based on deep learning, was used to quantify the number, velocity, displacement, and other parameters representing the migration capacity of dendritic cells. With the support of this system, the effective movement of dendritic cells toward tumor cells was assessed.

As immune cells explore known environments such as probes, the analysis of the environment could yield information about how human peripheral blood mononuclear cells (PBMCs) approach tumor cells. To monitor the interaction between PBMCs and tumor cells, Biselli et al. utilized OoC technology and reconstructed a tumor-on-a-chip that cocultured the PBMCs with HER2^+^ tumor cells (Figure 10(b)) [96]. Through a customized algorithm, the authors showed that the experimental conditions of time-lapse microscopy directly influenced the accuracy of the cell tracking algorithm. Based on the same chip, Comes et al. further investigated the impact of temporal and spatial resolutions on the reliability of OoC experimental results (Figure 10(c)) [97]. Using this method, the authors successfully obtained the kinematic characteristics of cells under different therapeutic conditions and revealed the efficacy of targeted therapy. This work also demonstrated the important role of OoCs as a bridge between biology and computer science in high-content image-based data extraction.

**Figure 11:**
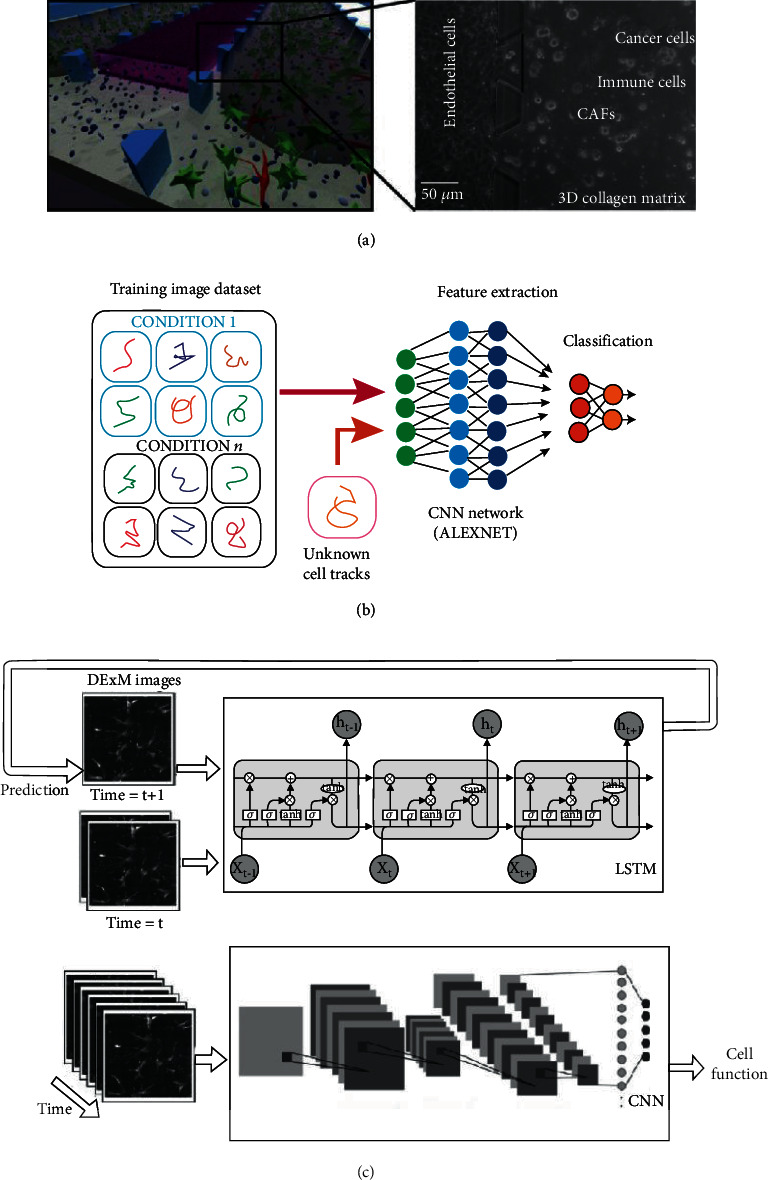
Deep learning in OoCs. (a) Tumor-on-a-chip approach. A central vascular compartment made of a monolayer of endothelial cells (pink), lateral chambers with 3D collagen hydrogels (gray) in which cancer cells (green), immune cells (blue), and CAFs (red) were embedded (reproduced with permission from Ref. [98]). (b) Schematic representation of the proposed method (reproduced with permission from Ref. [99]). (c) Differential expansion microscopy (DExM) was fed into an RNN architecture with LSTM memory blocks to predict the morphology of muscle cells and gain temporal DExM images. These images were then used by a CNN architecture for the prediction of cell function (reproduced with permission from Ref. [100]).

A major challenge in cancer research is an increase in the complexity of the tumor microenvironment. Related to the aforementioned examples, Nguyen et al., in the group mentioned earlier, built a more sophisticated HER2^+^ breast tumor microenvironment in the tumor-on-a-chip (Figure 11(a)) [98]. In addition to HER2^+^ breast cancer cells, endothelial cells and fibroblasts were cocultured to better replicate the microenvironment of the tumor tissue. Similar to previous studies, immune cells were applied, and CellHunter [95] was used to track the interactions between cells in OoCs. This integration of deep learning and OoCs enabled visualization and quantification of the complex dynamics of the tumor cells in the model. The results demonstrated that the tumor-on-a-chip was a powerful platform for the study of the interaction between immune cells and the tumor microenvironment, as well as the responses of the immune cell–tumor ecosystem to drug treatment.

The same group further optimized the algorithm and developed a novel deep learning tool called deep tracking [99]. The first step was to acquire a video of cells moving on an OoC platform. In the video, the trajectory images were collected under a range of circumstances. For a human expert, this step made the cell trajectories more conspicuous than the multichannel time-series approach. The second step utilized a pretrained CNN architecture, AlexNet, to classify the cell trajectories from the visual atlas of each experiment. The author tested deep tracking in two types of tumor-on-a-chip (Figure 11(b)). By using deep tracking, the addition of the immunotherapy drug trastuzumab was found to increase cancer–immune cell interactions. The high accuracy of the model illustrates the versatility of deep tracking. Normally, manually collected information is necessary in deep learning; however, the parameters in deep tracking algorithms are not tuned according to cyclic experiments. Therefore, we believe that by adding key parameters that are manually tuned by experts to the system, an even higher accuracy will be realized for deep tracking.

Substantial and energy metabolism are supported by skeletal muscles. It is important to simulate the physiological state *in vitro* because of the dynamic features of muscles. Jena et al. cultured primary human skeletal muscle cells in a 3D stretchable platform to generate a human muscle-on-a-chip [100]. In this work, the authors used an RNN architecture with LSTM memory blocks and the CNN architecture shown in Figure 11(c), where the algorithm is more complicated than deep tracking [99]. They inferred differences in position and morphology based on the time sequences of the images predicted by the RNN architecture. The authors further applied these images to a further CNN architecture. Using this deep learning framework, biochemical markers were successfully determined based on static and dynamic imaging of cells over time. This type of trained CNN architecture can also be used to judge the physiological status, contractile type, and performance of muscle cells, without expensive and complicated biochemical detection. Furthermore, this muscle-on-a-chip can provide a reference for early diagnosis and methods for the development of personalized medicine.

### 4.3. Key Applications of Deep Learning in OoCs

**Figure 12:**
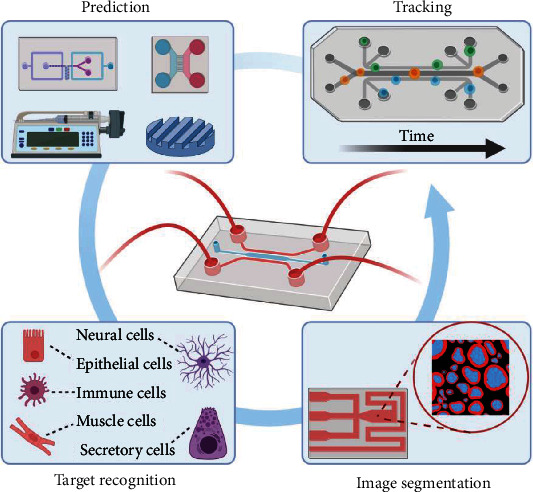
Key applications of deep learning in OoCs are prediction, target recognition, image segmentation, and tracking.

Through examples of the successful integration of deep learning and microfluidics, several key perspectives can be identified (Figure 12). In the project preparation stage, deep learning can be applied to the device design and material selection of OoCs, resulting in OoCs more suitable for the particular application. In addition, owing to the increasing popularity of multiple-cell cultures in OoCs, deep learning can also be used for robust discrimination of cell populations.

In some OoCs, it is important to segment the parts of the images with special significance and extract the relevant features to provide reliable results for the analysis of experimental data. For instance, the brain tumor-on-a-chip reported by Yi et al. printed brain tumor cells in 3D, where endothelial cells were cultured surrounding the tumor cells [101]. Several key actions in this study involved the separation of endothelial cells, neoplastic cells, and tumor stem cells with different fluorescent labeling. An automatic image-based system that can extract different segments of the tumor-on-a-chip will facilitate the analysis of anticancer drug therapy. However, the complexity of the image itself and issues such as inhomogeneity and individual differences in the segmentation process make it difficult for traditional machine learning methods and typical neural networks to achieve pixel-level segmentation of images.

In 2015, Long et al. first applied fully convolutional networks (FCNs), which can accept input images of arbitrary size and perform pixel-by-pixel classification of images [102]. The U-Net model is a modified FCN structure. It is named for its structure, which is shaped like the letter U, and is widely used in image semantic segmentation [68]. Zaimi et al. used a U-Net architecture to achieve pixel-level segmentation of axons, myelin sheaths, and backgrounds in images [103]. The same system could be applied to the pixel-level segmentation of images obtained from OoC. The type of CNN architecture used in the downsampling process can also be changed in semantic image segmentation. Lim et al. reconstructed all pixels of red blood cells and greatly improved the image quality and superresolution capability of the model [104]. This study contained two highlights: one was the generation of digital phantoms as input, which overcame the lack of ground truth and eliminated the introduced distortions. The second was the presentation of a U-Net architecture with a skip connection between input and output, which mitigated the vanishing gradient problem during training and increased its performance. Developers have now programmed a plug-in for single-cell segmentation in the ImageJ software that allows people who are unfamiliar with deep learning to use U-Net to analyze data on a personal computer [105]. In addition to the U-Net model, models, such as DeepCell [106], CDeep3M [107], and CellProfiler [108], have been used to achieve pixel-level segmentation of cellular images.

Finally, the real-time visualization of cell morphology and trajectory is crucial for medical research. Zhao et al. combined a classic U-Net architecture with glass–air Anderson localizing optical fibers (GALOFs) [109]. This imaging system, named Cell-DCNN-GALOF, could transfer high-quality images in real time. Furthermore, the image reconstruction process was remarkably robust with respect to various depths, temperature variations, and fiber bending. Most importantly, the system showed a unique transfer-learning capability when cells with different morphologies and classes were examined. This means that the system could be trained with cell images from OoCs. Cell trajectory monitoring could also be realized through Cell-DCNN-GALOF in real time. By automatically detecting their trajectory and quantifying relevant movement data, deep learning can also provide auxiliary references to quantify experimental results and develop new experimental models for OoCs. Therefore, we believe that OoCs will transform traditional approaches, which rely heavily on manual data processing and operation, into a highly automatic system by means of deep learning.

## 5. Summary and Prospects

### 5.1. Future Applications of OoCs

In recent years, deep learning has completely changed many traditional industrial systems. Moreover, researchers in different fields are trying to integrate deep learning technology into their respective fields. In particular, many research advances have shown the promise of the integration of OoC technology and deep learning. The main reasons for this are as follows.

First, the open-source projects implementing deep learning have gradually improved, including the open-source code based on TensorFlow, Pytorch, and Keras frameworks. These allow researchers to quickly reproduce open-source engineering code in different fields and apply it to their own specific tasks. Second, novel and better deep learning models with excellent performance are constantly emerging. These will ultimately be applied to the rapid development of industries that are extensively integrated with deep learning. Lastly, the multitype data generated by OoC experiments are not only complex but also large in quantity. Deep learning technology can be introduced to simplify the labor-intensive data analysis and feature extraction steps, alleviate the huge challenges brought by massive biomedical big data, and solve tasks that were previously considered infeasible.

#### 5.1.1. Organelle Segmentation and Tracking in OoCs

Current applications of deep learning in cell biology mainly focus on the morphological changes of whole cells; however, the analysis of subcellular structures or organelles can provide additional and important information [110, 111]. Very recently, Lefebvre et al. developed a shallow machine learning algorithm-based software package named Mitometer that can rapidly segment and track cellular mitochondria from both images and videos [112]. This provides a new and exciting research direction for AI in cell biology. As mentioned previously, deep learning captures the inherent features of data in a more efficient and accurate way than traditional machine learning. We therefore believe that deep learning-based organelle segmentation and tracking can generate comparatively richer information on the cell/tissue behavior in the OoCs.

#### 5.1.2. OoC Tissue Mechanical Force Control

Tissue mechanical force control is one of the most important features of OoCs. For example, in the case of a lung-on-a-chip, human airway epithelial cells were cultured in a computer-controlled two-phase microfluidic system. The system can simulate the propagation and rupture of fluid embolism that occurs during airway injury in obstructive lung disease [113]. As the timing of applying the tissue mechanical force is critical, it is necessary to develop an automatic instrument based on the cell morphology and microenvironment. Deep learning detects cell processes and biomarkers over time without affecting cell viability; therefore, it allows the monitoring of the performance of the entire system in real time [114]. By integrating deep learning with OoC, the system can exploit the potential to automatically regulate and control various functional parameters of OoCs.

#### 5.1.3. Drug Screening on OoCs

OoCs have been widely explored as *in vitro* human-related disease models, which can be an excellent platform for the study of pharmacokinetics, drug toxicity, and pharmacology. For example, Boos et al. tested embryoid bodies, influenced by human liver metabolites, in an organoid system [115]. The platform provides a promising tool that more comprehensively reflects physiological processes in *in vitro* tests, thus increasing the predictive power of adverse drug effects. Thus, OoCs can be used to investigate embryotoxicity. Several examples [116, 117] have also illustrated the potential of the deep learning-based system in the accurate prediction of the efficacy and toxicity of therapeutics. Therefore, the predictive capabilities of deep learning and OoC integration are a promising and important tool for future drug discovery.

#### 5.1.4. Rare Disease-on-Chips

OoC technology has been widely utilized to build various *in vitro* disease models [1]. However, in recent years, the development of new drugs for rare diseases has been greatly hampered by the scarcity of suitable preclinical models for clinical trials [118, 119]. OoC-based rare disease models generate important real-time data that cannot usually be observed in *in vivo* or clinical samples. These data can be further analyzed by deep learning in real time, thus enabling the analysis of changes in the development of such diseases at the molecular level and ultimately obtaining the specific mechanisms of disease occurrence.

#### 5.1.5. Human-on-Chips

Human-on-chips, a further development of OoCs, consist of interconnected compartments. Each compartment (i.e., an OoC) contains specific cell types that represent different organs. All compartments are connected by a microfluidic circulation system [120], which is highly modular in nature. The features of different compartments can be extracted through deep learning, and the output of the previous compartment can be utilized as the input to the next compartment. The linkage of multiorgan tissue system interaction is of great benefit to physiologically based pharmacokinetic models, quantitative system pharmacology, and other models [1]. Recently, a robotic interrogator automatically cultured, perfused medium, and linked fluidic systems, to maintain the viability and organ-specific functions of eight vascularized two-channel organ chips for three weeks [121]. In addition, a high-throughput human-on-a-chip system was formed on a medium circulation platform, which enabled parallelized multiorgan experiments [122]. Furthermore, human-on-chips have already been used in the study of intestinal absorption, hepatic metabolism, and the activity of breast cancer drugs [123]. In the future, through the analysis of multiple data of each OoC (such as cell growth, differentiation, and metabolism) using deep learning, OoCs can be combined into a highly integrated and controllable microfluidic regulatory system, thus achieving self-intelligent regulation of OoCs. Indeed, subsequent work and collaborations are still required to push the development of the integration of multiple OoCs and deep learning.

### 5.2. Future Challenges in Deep Learning

Although deep learning technology has excellent performance in feature representation and data mining, its internal mechanism and calculation strategy still need to be optimized for specific applications. Therefore, the development of a highly automated OoC system to provide a convenient, reliable, and integrated intelligent platform for researchers is the main development direction and challenge in the future.

#### 5.2.1. Data Processing

At present, the ability to acquire experimental data on OoCs has been greatly improved, and massive amounts of big data have been accumulated. However, reducing the huge cost of manual labeling and automatically mining and refining the inherent characteristics of massive data are key challenges that urgently need to be overcome. The following aspects of this can be explored.

1. *Data Augmentation*. The most advanced methods such as GAN and unsupervised data augmentation (the latest unsupervised data augmentation method proposed by Google has a better application in the field of natural scene images) can be introduced to generate simulation data with real data characteristics, expand the capacity of high-value data samples, and reduce the cost of data acquisition.
2. *Automatic Data Annotation*. The development of automatic data annotation algorithms and tools enables the automatic labeling of massive unlabeled data, reducing the huge cost of manual labeling, and improving the efficiency of labeling and development.
3. *Semisupervised Learning*. Semisupervised learning [124] can be introduced to reduce the dependence on massive labeled data. Active learning [125, 126], a special learning method in semisupervised learning, uses a small amount of labeled data; its effect is equal to supervised learning. Furthermore, self-supervised learning, as an unsupervised learning approach, only uses unlabeled data to learn a feature extractor, whose performance is optimized by pretraining and fine-tuning [127].
4. *Transfer Learning*. Transfer learning can also be used as an excellent technical means to reduce dependence on a large volume of labeled data. The network is pretrained using the labeled data from other domains. Then, the fine-tuning of the network can be completed with a small amount of labeled data in a specific domain.

#### 5.2.2. Algorithm Upgrading

1. *Customized Design of Deep Learning Models*. Facing the diverse application requirements in the field of OoCs, the deep learning models that are used in the field of natural optical images cannot be directly applied. Therefore, customized designs are required, and improvements must be made in terms of the model architecture and deep network layout.
2. *Automatic Network Design*. For specific application scenarios of OoCs, neural architecture search technology can be used to realize the automatic construction and the optimal intelligent design of deep models, which avoids the complexity and limitations of traditional deep models design based on expert experience.
3. *Iterative Upgrade of Deep Models*. Facing the increasingly updated OoC data, deep learning models usually have the problem of “catastrophic forgetting.” Therefore, enabling the deep learning model to handle new data and maintain robust performance requires further exploration of the iterative upgrade technology of deep learning models.
4. *Interpretability*. Deep learning models usually have the problem of the “black box effect”; that is, the internal mechanism is not clear. This limits the understanding of the underlying mechanism and interaction principle of the specific OoC, which then lacks interpretability and reliability. Therefore, there is an urgent need to develop interpretable deep learning technology to transform the “black box” of deep learning into a “white box” and enable meaningful physical explanations from a biological point of view.
5. *Model Compression and Acceleration*. For online scene applications, the accuracy and inference speed of the model need to be balanced. Therefore, it is necessary to thoroughly study the compression and acceleration technology of deep learning models, and under the premise of ensuring accuracy, compress the model volume to the extent possible to improve the inference speed.

#### 5.2.3. Computing Capability

At present, the application of deep learning models is mainly based on computing hardware including GPU, CPU, FPGA, and other devices. The GPU and CPU are used for offline training of deep learning models. In particular, owing to the efficient computing power of the GPU, it has become the main hardware used for deep learning model training, including many products made by NVIDIA. FPGAs are mainly used in online applications and are edge devices for real-world applications.

With the development of distributed technology, current devices such as GPUs and FPGAs have been able to meet the application of deep learning models. In the future, it will be necessary to combine hardware resources to carry out research to improve the inference efficiency of deep learning models and enhance the reliable transfer and rapid deployment of models to meet additional application scenarios.

## Figures and Tables

**Table 1:** Summary of different applications for deep learning in microfluidics and deep learning in OoCs.

Application	Experiment		Network			Function	Refs
	Device design	Subject	Input	Architecture	Output		
Deep learning in microfluidics	A microfluidic device with three capillary tubes	The generated microdroplet in a T-junction microfluidic system	Four numbers affect the size of microdroplet	An ANN architecture	Length of the droplet and the diameter of the junction	Predict the size of microdroplet at the exit of the T-junction according to different parameters	[71]
	Two pressure sensors and a single microchannel filled with a liquid metal	Microfluidic soft sensors	An analog voltage	An RNN with an attention module	Pressure estimation and localization	Estimate both pressure magnitude and location while considering the hysteresis problem	[72]
	A fluid flow shape model decided by micropillars	Flow sculpting	The top-half image of a microchannel shape	A CNN architecture	Corresponding pillar sequences	Make predictions and deliver comparable designs for flow sculpting	[73]
	Two microfluidic devices with four culture channels	*Pseudomonas aeruginosa* bacteria	Spectrum images that convert from original images via FFT	An AlexNet architecture	Pixel count in the spectrum images	Recognize the regional concentration change of the cultured bacteria	[91]
	A plastic slide with physical channels in medium	Bone marrow from mice tibiae and ilia	Long-term and time-lapse microscopy cell patches	A CNN-RNN architecture	Cell lineage score	Predict the lineage choice of stem cells' progeny	[92]

Deep learning in OoCs	A microfluidic device composed of a central immune chamber and two tumor chambers	Interferon-*α*-conditioned dendritic cells (IFN-DCs)	Time-lapse images that record cells' trajectory in 3D tumor spaces	An unsupervised image analysis algorithm, Cell Hunter	Parameters that characterize IFN-DC behavior toward cancer cells	Track immune cell-tumor interactions in real time	[94]
	A microfluidic device composed of six reservoirs and four chambers	Three groups of human PBMCs	Data that collect by a microfluidic platform and time-lapse video	Cell Hunter	Some trajectories of specific cell	Track the migration and the interactions of human PBMCs toward tumor cells	[96]
	A microfluidic device with 3D biomimetic hydrogels inside microchambers	HER2^+^ breast cancer BT474 cell line and PBMCs	An atlas of videos at varying spatial-temporal resolutions	Cell Hunter	A set of kinematic and interaction descriptors	Describe the motility and interaction at varying spatial-temporal resolutions	[97]
	A 3D coculture microfluidic device with a central vascular compartment and two lateral chambers	BT474 cell line of HER2^+^ breast cancer, the breast CAF cell line Hs578T and PBMCs	Time-lapse videos and images that reconstructed in 3D	Cell Hunter	Parameters that record the interaction of a single cancer cell with all the PBMCs	Characterize the responses to the drug and dissect the roles of immune cells and fibroblasts	[98]
	A microfluidic device with 3D biomimetic gels	BT474 cell line of HER2^+^ breast cancer and PBMCs	Video sequence of cells	Cell Hunter and a CNN architecture	Atlas of experimental cell tracks and type	Discover hidden messages within cell trajectories for cancer drug treatments	[99]
	A stretchable micropatterned 3D human skeletal muscle platform	Human skeletal muscle cells and myogenic stem cells	Morphological image of skeletal muscle cells	A CNN-RNN architecture	Temporal prediction and cell function of muscle cells	Judge the physiological status, contractile type and performance of muscle cells more easily	[100]
